# Harnessing *Bacillus velezensis* for enhanced protease biosynthesis: optimization, purification, and kinetic profiling

**DOI:** 10.1186/s12866-026-04782-6

**Published:** 2026-03-02

**Authors:** Saied N. Fergany, Salem S. Salem, Mohamed A. Abdel-Naby, Tarek M. Abdelghany, Samah H. Abu-Hussien

**Affiliations:** 1 Botany and Microbiology Department, Faculty of Science, Al-Azhar University, Nasr City, Cairo, 11884 Egypt; 2 Chemistry of Natural and Microbial Products Department, National Research Centre, Dokki, Giza, Egypt; 3 Agricultural Microbiology Department, Faculty of Agriculture, Ain Shams University, Cairo, 11241 Egypt

**Keywords:** Bacillus velezensis, Protease production, Response surface methodology, Box-Behnken design, Enzyme optimization, Microbial screening

## Abstract

Microbial proteases represent critical biocatalysts for industrial applications, yet optimizing their production remains challenging. This study isolated and characterized a high-yield protease-producing bacterium from environmental sources and systematically optimized production parameters. Among 124 bacterial isolates screened from six environmental sources, poultry waste exhibited the highest microbial diversity, yielding 63 morphologically diverse isolates. The most potent proteolytic isolate, P15, was identified through 16 S rRNA gene sequencing as *Bacillus velezensis* AZH3S (GenBank accession PX417380), sharing 99% sequence similarity with *B. amyloliquefaciens*. Initial screening of eight culture media identified glucose-casein-yeast extract medium as optimal, producing 442 U/mL protease activity. Time-course analysis revealed maximum enzyme production at 24 h (420 U/mL), with yield coefficients increasing to 480 U/g biomass in late stationary phase. Carbon and nitrogen source optimization demonstrated that starch (470 U/mL) and peptone (480 U/mL) were superior substrates. Response surface methodology employing Box-Behnken design across five variables (starch, peptone, pH, temperature, agitation speed) generated a highly predictive model (R² = 0.99) identifying agitation speed as the most influential parameter. Optimized conditions (9.84 g/L starch, 5.46 g/L peptone, pH 7.5, 33.1 °C, 244 rpm) achieved 1160.3 ± 9.20U/mL protease activity, representing a 2.80-fold improvement over initial production. Three-step purification achieved 5.82-fold enrichment with 32.0% yield. The purified enzyme exhibited a molecular mass of approximately 20 kDa with exceptional substrate affinity (Km = 0.207 mM, Vmax = 39.8 µmol/min), following classical Michaelis-Menten kinetics.These findings establish a robust bioprocess for industrial-scale protease production with significant biotechnological potential.

**Supplementary Information:**

The online version contains supplementary material available at 10.1186/s12866-026-04782-6.

## Introduction

Proteases (EC 3.4) represent approximately 60% of the global enzyme market with projections exceeding $6.3 billion by 2027 [1]. These biocatalysts find applications in detergent manufacturing [2], pharmaceutical interventions, food processing, and leather industries [3]. The escalating demand for cost-effective, stable proteases has intensified research toward discovering novel microbial strains and optimizing production parameters [4].

Microbial enzymes are favored for industrial production due to superior yields, shorter production cycles, and amenability to controlled fermentation [5–7]. Submerged fermentation (SmF) enables precise parameter control suitable for statistical optimization [8–12]. However, protease productivity exhibits high sensitivity to pH, temperature, dissolved oxygen, and nutrient availability, making optimization essential [13].

Response surface methodology (RSM), particularly Box–Behnken design (BBD), has revolutionized bioprocess development by generating mathematical models describing relationships between process parameters and enzyme yields while reducing experimental runs. Despite these advances, a critical gap persists: most studies have either generalized enzyme optimization without protease-specific focus or targeted co-production of protease alongside other enzymes [9]. Co-production strategies often lead to metabolic competition and diminished yields. Systematic optimization of protease production as an independent bioprocess remains critically underexplored [14–16].

Members of the genus *Bacillus* are widely used in industrial enzyme production owing to their efficient secretion systems, genetic tractability, robustness under fermentation conditions, and, for certain well-characterized strains, recognized safety profiles (GRAS); however, GRAS status is strain-specific and industrial adoption depends on a combination of these factors [17–21]. *Bacillus velezensis*, a recently reclassified member of the *B. amyloliquefaciens* group, possesses extensive genes encoding subtilisin-like serine proteases with broad substrate specificity. Despite these advantages, systematic investigations optimizing protease production from *B. velezensis* using advanced statistical designs remain limited [22–24].

Carbon and nitrogen sources critically influence protease biosynthesis by affecting cellular metabolism and enzyme secretion [17, 18, 25]. Complex organic nitrogen sources often function as both nutrients and inducers, while carbon sources modulate catabolite repression [25, 26]. Physical parameters including agitation, temperature, and pH orchestrate regulatory networks affecting growth kinetics and oxygen transfer [27, 28]. Elucidating these interactions through RSM-based designs is essential for developing scalable bioprocesses.

Environmental bioprospecting identifies microorganisms with superior enzymatic capabilities. Poultry waste, agricultural soils, and animal dung harbor bacterial populations with specialized proteolytic systems optimized for protein degradation. Beyond yield optimization, biochemical characterization including molecular weight and kinetic parameters (Km, Vmax) determines enzyme suitability for industrial applications [21, 29]. The novelty of this investigation lies in its integrated approach combining environmental screening, molecular identification, and dedicated statistical optimization of protease production as an independent bioprocess. By systematically evaluating nutritional and physicochemical factors through Box–Behnken design, this work reveals complex interactions typically missed by conventional approaches. Specific objectives: (i) isolate and screen proteolytic bacteria from diverse environmental sources; (ii) identify the most potent isolate through 16 S rRNA sequencing; (iii) evaluate effects of media, carbon sources, and nitrogen sources on protease production; (iv) construct a quadratic regression model using BBD to optimize five fermentation parameters; (v) validate the optimized model experimentally; (vi) purify and characterize the protease including molecular weight and kinetic parameters. The findings establish an economically viable bioprocess for high-yield protease production from *Bacillus velezensis*, addressing industrial needs for sustainable biocatalytic solutions [30, 31].

## Materials and methods

### Chemicals and reagents

All chemicals were of analytical grade. Casein, peptone, tryptone, yeast extract, and skim milk powder were obtained from HiMedia Laboratories (Mumbai, India). Starch, glucose, and glycerol were purchased from Merck (Darmstadt, Germany). Ammonium sulfate, Tris-HCl, and Folin-Ciocalteu reagent were from Sigma-Aldrich (St. Louis, MO, USA). DEAE-cellulose and Sephadex G-100 were from GE Healthcare (Uppsala, Sweden). Molecular biology reagents including PCR Master Mix and DNA extraction kits were from Qiagen (Hilden, Germany) and Thermo Fisher Scientific (Waltham, MA, USA).

### Sample collection and processing

Environmental samples (*n* = 3 per site) were aseptically collected from six distinct sources: poultry waste, agricultural soil (0–15 cm depth), coastal sand, seawater, cattle dung, and sheep/goat dung. All samples were transported on ice (4 °C) and processed within 24 h of collection. For solid matrices, 10 g of material was homogenized in 90 mL of sterile physiological saline (0.85% w/v NaCl) by vortexing for 5 min, followed by serial dilution (10⁻¹–10⁻⁶). Seawater samples (10 mL) were directly subjected to serial dilution.

### Isolation and characterization

Aliquots (100 µL) from appropriate dilutions were spread-plated in triplicate onto skim milk agar (SMA: 5 g/L peptone, 2.5 g/L yeast extract, 1 g/L glucose, 15 g/L agar, 100 mL/L skim milk, pH 7.0) and incubated at 37 °C for 48–72 h. Colonies with clear hydrolysis zones were selected based on proteolytic index (PI = total zone diameter/colony diameter) ≥ 2.0 and purified by three successive subcultures. Pure isolates were Gram-stained and examined at 1000× magnification to determine cell morphology and Gram reaction. Cultures were preserved at 4 °C on nutrient agar slants and at −80 °C in 20% glycerol [32].

### Phenotypic and molecular identification of isolate P15

#### Phenotypic characterization of isolate P15

Phenotypic characterization included assessment of colony features on nutrient agar incubated at 37 °C for 24–48 h, noting color, form, elevation, margin, and texture. Cellular morphology was examined by Gram staining using standard procedures. Endospore formation was evaluated by the Schaeffer–Fulton method, and stained smears were observed under oil immersion (1000×). Motility was assessed using both the hanging drop method and semi-solid agar (0.4%) stab inoculation. Biochemical tests included catalase, oxidase, starch and casein hydrolysis, gelatin liquefaction, citrate utilization, and nitrate reduction following established protocols. Growth performance was examined in nutrient broth across a range of temperatures (25–45 °C), pH values (5.0–9.0), and NaCl concentrations (0–10% w/v) [33].

#### Molecular identification of isolate P15

Genomic DNA was extracted using DNeasy Blood & Tissue Kit (Qiagen, Germany) and quantified by NanoDrop spectrophotometry (A₂₆₀/A₂₈₀). The 16 S rRNA gene was amplified using universal primers 27 F (5′-AGAGTTTGATCCTGGCTCAG-3′) and 1492R (5′-TACGGCTACCTTGTTACGACTT-3′). PCR reactions (50 µL) contained: 25 µL 2× PCR Master Mix (Thermo Fisher Scientific), 1 µL each primer (10 µM), 2 µL template DNA (50 ng), and 21 µL nuclease-free water. Thermal cycling: initial denaturation 95 °C/5 min; 35 cycles of 95 °C/30 s, 55 °C/30 s, 72 °C/90 s; final extension 72 °C/10 min. PCR products were visualized on 1.5% agarose gel, purified using QIAquick Gel Extraction Kit (Qiagen), and sequenced bidirectionally (Macrogen Inc., South Korea). Sequences were trimmed and assembled using BioEdit v7.0.4, aligned with NCBI GenBank sequences using ClustalW v4.5.1, and analyzed via BLASTn. Phylogenetic analysis was performed using MEGA v11 with neighbor-joining method, Kimura 2-parameter model, and 1000 bootstrap replicates [7].

### Optimization of protease production

#### One-Factor-at-a-Time (OFAT) screening of variables influencing protease production

##### Time course of protease production

Standardized inoculum of P15 isolate (OD₆₀₀ = 0.6, ~ 2 × 10⁷ CFU/mL) was inoculated at 5% (v/v) into 100 mL casein–glucose medium and incubated at 37 °C, 120 rpm for 48 h. Ten-milliliter samples were collected every 6 h, centrifuged (10,000 rpm, 15 min, 4 °C), and supernatants assayed for protease activity using casein hydrolysis with tyrosine standard (U/mL). Cell dry weight (CDW) was determined from pellets dried to constant weight at 80 °C. All assays were performed in triplicate. Specific production rate (µp, h⁻¹) was calculated as µp = (ln P − ln P₀)/(t − t₀), where P and P₀ represent protease concentrations at times t and t₀ [34].

#### Effect of different media

To identify optimal culture medium, eight broth formulations (Media 1–8) were evaluated. Each medium (50 mL in 250 mL flasks) was sterilized and inoculated with 2.5 mL standard inoculum (OD₆₀₀ = 0.1, ~ 7.0 × 10⁵ CFU/mL). Media represented varying carbon/nitrogen complexity: simple defined media (glucose-based), complex organic media (peptone/tryptone-rich), and protein-rich inductive media (casein/gelatin-based) to evaluate nutrient-induced protease expression and catabolite repression effects. Initial pH was adjusted to 7.0, and flasks were incubated at 37 °C, 120 rpm for 24 h. Ten-milliliter samples were collected for protease quantification [35].

### Influence of nutritional parameters on protease production

#### Experimental design and fermentation conditions

Fermentation experiments were conducted in 250 mL Erlenmeyer flasks containing 50 mL of TGY medium (medium 6) as the basal medium. Each flask was inoculated with 2.5 mL of standardized inoculum (OD₆₀₀ = 0.6, approximately 2 × 10⁷ CFU/mL) of P15 isolate and incubated at 30 °C for 24 h in a rotary shaker (Lab-line Ltd., USA) at 120 rpm, except when temperature or agitation effects were specifically investigated. Following incubation, biomass concentration and protease activity were determined. All experiments were performed in triplicate, and mean values were reported [34].

Fermentation experiments were conducted in 250 mL flasks containing 50 mL TGY medium (medium 6) inoculated with 2.5 mL standardized inoculum (OD₆₀₀ = 0.6, ~ 2 × 10⁷ CFU/mL) and incubated at 30 °C, 120 rpm for 24 h, except when temperature or agitation effects were investigated. All experiments were performed in triplicate. Carbon source screening: Glucose in TGY medium was replaced with equimolar carbon equivalents of nine substrates: starch, mannitol, glycerol, sorbitol, maltose, lactose, sucrose, mannose, and fructose, with glucose as control. Medium pH was adjusted to 7.0 before sterilization. Complex carbohydrates and polyols were filter-sterilized and added aseptically. Following identification of optimal carbon source, concentration optimization was performed at 0, 8, 10, and 15 g/L. Nitrogen source screening: Thirteen nitrogen sources were evaluated: casamino acids, soybean meal, peptone, gelatin, tryptone, urea, yeast extract, casein, NaNO₃, NH₄NO₃, NH₄Cl, (NH₄)₂SO₄, and KNO₃, normalized to equivalent nitrogen content (14% w/w). Optimal nitrogen source was evaluated at 5, 10, and 15 g/L. Biomass yield, protease activity, volumetric productivity (U/mL/h), and specific yield coefficient (U/g biomass) were quantified [34].

### Statistical optimization of protease production using P15 isolate by Box-Behnken design

#### Experimental design and statistical analysis

A five-factor, three-level Box-Behnken Design (BBD) was employed to optimize protease production using Design-Expert software (version 13, Stat-Ease Inc., Minneapolis, MN, USA). The independent variables starch concentration (5–10 g/L), peptone concentration (5–10 g/L), pH (5–9), temperature (25–40 °C), and agitation speed (100–250 rpm) were evaluated across 46 experimental runs (Table 1 and Table S1), including six center-point replicates to estimate experimental error [36]. Biomass during RSM experiments was determined gravimetrically by filtering culture samples through pre-weighed 0.45 μm membranes, washing with distilled water, and drying at 80 °C to constant weight.

**Table 1:** Independent variables and experimental design matrix for Box-Behnken optimization

Factor	Variable	Unit	Coded Level		
			–1	0	+ 1
A	Starch	g/L	5.00	7.50	10.00
B	Peptone	g/L	5.00	7.50	10.00
C	pH	—	5.00	7.00	9.00
D	Temperature	°C	25.00	32.50	40.00
E	Agitation	rpm	100.00	175.00	250.00

### Enzyme extraction, assay, purification, and kinetic characterization

#### Crude enzyme extraction

Following fermentation, culture broths were centrifuged at 10,000×g for 10 min at 4 °C to separate the supernatant containing extracellular proteases. Cell pellets were retained for dry biomass determination, while clarified supernatants were stored at 4 °C and assayed for enzyme activity within 24 h [34].

#### Protease activity assay

Proteolytic activity was determined using modified casein-Folin method. One milliliter diluted enzyme solution was mixed with 5 mL casein solution (0.65% w/v in 50 mM potassium phosphate buffer, pH 7.0) and incubated at 37 °C for 10 min. Reaction was terminated with 5 mL of 0.11 M trichloroacetic acid (TCA), incubated 30 min at room temperature, and centrifuged (5,000×g, 15 min). Supernatant (2 mL) was mixed with 5 mL of 0.5 M sodium carbonate and 1 mL Folin-Ciocalteu reagent (1:1 dilution), incubated 30 min, and absorbance measured at 660 nm against reagent blank. Tyrosine standard curve (0–1000 µg/mL) was prepared in triplicate. One unit (U) of protease activity was defined as enzyme releasing 1 µg tyrosine per minute under specified conditions [34].

#### Protease purification

Culture supernatants from 24 h fermentations were subjected to three-step purification. Proteins were precipitated by ammonium sulfate fractionation (60–90% saturation), collected by centrifugation (15,000×g, 30 min, 4 °C), and dialyzed overnight against 20 mM Tris-HCl buffer (pH 7.5) at 4 °C using 12–14 kDa MWCO membranes. Dialyzed samples were applied to DEAE-cellulose column (2.5 × 18 cm) pre-equilibrated with 20 mM Tris-HCl buffer (pH 7.5) and eluted with linear NaCl gradient (0–1 M) at 1 mL/min. Active fractions were pooled and concentrated by ultrafiltration (10 kDa MWCO). Concentrated samples underwent gel filtration on Sephadex G-100 column (2.5 × 60 cm) equilibrated with 50 mM potassium phosphate buffer (pH 7.0) containing 150 mM NaCl at 0.3 mL/min. Active fractions were pooled, concentrated, and stored at − 20 °C in 20% (v/v) glycerol. All operations were performed at 4 °C with specific activity monitored at each step [2].

#### SDS-PAGE analysis

Purified protease samples were analyzed by sodium dodecyl sulfate-polyacrylamide gel electrophoresis (SDS-PAGE) under denaturing conditions. Protein samples (5–10 µg) were mixed with 2× Laemmli sample buffer (4% SDS, 10% β-mercaptoethanol, 20% glycerol, 0.004% bromophenol blue, 0.125 M Tris-HCl, pH 6.8) and heated at 95 °C for 5 min. Electrophoresis was performed on 12% polyacrylamide gels using a Mini-PROTEAN system (Bio-Rad, USA) at 80 V for stacking and 120 V for separation. Gels were fixed in 50% methanol/10% acetic acid, stained with 0.1% Coomassie Brilliant Blue R-250 in 50% methanol/10% acetic acid for 1 h, and destained in 40% methanol/10% acetic acid. Molecular weight was estimated using prestained protein ladder (GeneDirex PM008-0500, 10–250 kDa). Gels were imaged using a ChemiDoc imaging system (Bio-Rad, USA) [37, 38].

### Determination of kinetic parameters

Kinetic constants (Km and Vmax) of the purified protease were determined using casein as substrate at concentrations ranging from 0.1 to 2.5 mM in 50 mM potassium phosphate buffer (pH 7.0). Reaction mixtures containing varying substrate concentrations and appropriately diluted enzyme were incubated at 37 °C for 10 min. Reactions were terminated by adding 5 mL of 0.11 M TCA, and released tyrosine equivalents were quantified spectrophotometrically at 660 nm using the Folin-Ciocalteu method. Initial velocities were calculated from the linear portion of product formation curves. Kinetic parameters were determined by non-linear regression fitting to the Michaelis-Menten equation using GraphPad Prism software (version 9.0). Lineweaver-Burk plots were constructed by plotting 1/V versus 1/[S] to verify data linearity and calculate Km and Vmax from the x-intercept (–1/Km) and y-intercept (1/Vmax), respectively. All assays were performed in triplicate [8].

#### Proteases production parameters

Productivity (P) = Amount of proteases produced (UmL)/fermentation time (h) = U/mL/h. Proteases yield coefficient relative to biomass (Yp/x) (U/g) = Amount of proteases produced (U/mL)/amount of biomass (g/L). Specific activity of the sample was calculated by dividing the enzyme units (U) on the total protein content. Specific activity = Total enzyme units (U)/Total protein (mg/mL). The activity of the sample is expressed in units (U) and where ever necessary as specific activity (U/mg) [8].

### Statistical analysis

The data were subjected to one-way ANOVA, and differences among groups were evaluated using Tukey’s post-hoc test in SPSS version 12. A significance threshold of *P* < 0.05 was applied. Additionally, all experimental samples and datasets were analyzed using Design Expert 12 statistical software (https://www.statease.com/software/design-expert).

## Results and discussion

### Screening of proteolytic bacterial isolates based on source, number, and morphological characteristics

**Fig. 1:**
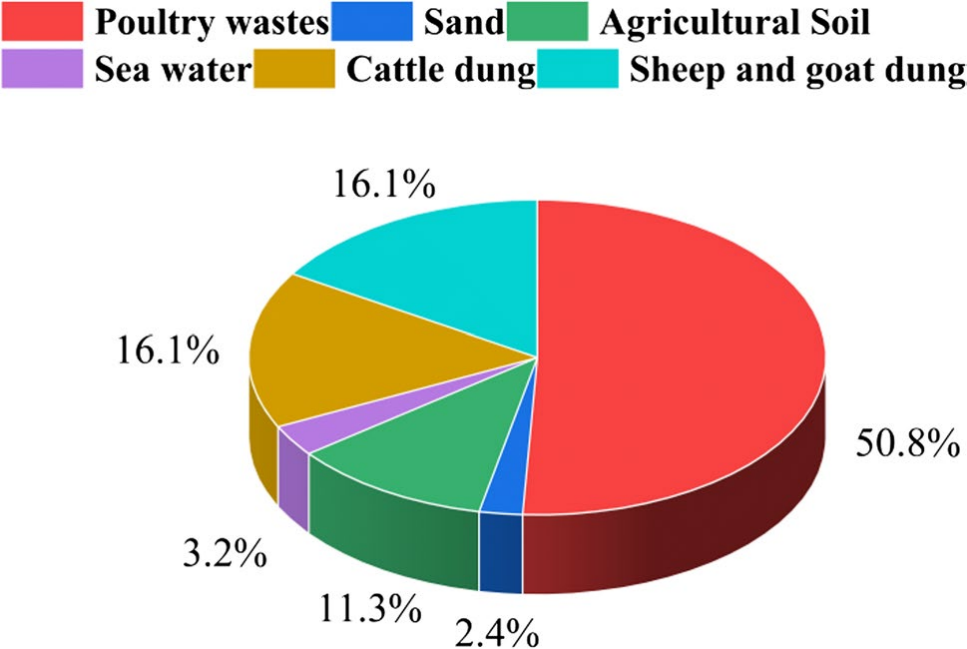
Distribution of proteolytic bacterial isolates from six environmental sources

From six environmental sources, 124 proteolytic bacterial isolates were recovered (Fig. 1), with poultry waste yielding the most (n = 63, 50.8%), followed by cattle and sheep/goat dung (n = 20 each, 16.1%), agricultural soil (n = 14, 11.3%), seawater (n = 4, 3.2%), and sand (n = 3, 2.4%) (p < 0.001). Six morphotypes (Gram-negative/positive cocci, rods, spirals) were identified. Poultry waste exhibited highest diversity (H’ = 1.58) with all morphotypes present, while ruminant dung showed no diversity (H’ = 0), containing only Gram-positive rods. Moderate diversity characterized agricultural soil (H’ = 0.95), seawater (H’ = 1.04), and sand (H’ = 1.10). Mean proteolytic index was highest in poultry waste (3.8 ± 1.2), significantly exceeding sand (2.6 ± 0.4, *p* < 0.01) and seawater (2.4 ± 0.3, *p* < 0.001). High nitrogen and protein content in poultry waste favored diverse morphotypes, whereas ruminant dung selected spore-forming *Bacillus* sp. adapted to low pH and thermophilic conditions. Nutrient scarcity in sand and seawater limited isolate recovery [39, 40].

#### Phenotypic identification

Based on proteolytic index screening (Table 2), isolate P15 from poultry waste exhibited the highest proteolytic activity (PI = 5.8). Phenotypic characterization revealed Gram-positive, rod-shaped, endospore-forming bacteria forming cream-colored, irregular, flat colonies with undulate margins on nutrient agar. Biochemical tests showed positive results for catalase, starch hydrolysis, gelatin liquefaction, and citrate utilization, with negative oxidase reaction (Table 3).

**Table 2:** Phenotypic and biochemical characteristics of isolate P15

Characteristic	Result
Morphology	
Cell shape	Rod
Gram reaction	Positive
Endospore formation	Positive (central/subterminal)
Motility	Positive
Colony morphology	
Color	Cream
Form	Irregular
Elevation	Flat
Margin	Undulate
Biochemical tests	
Catalase	Positive
Oxidase	Negative
Starch hydrolysis	Positive
Gelatin liquefaction	Positive
Citrate utilization	Positive
Casein hydrolysis	Positive
Nitrate reduction	Positive
Growth conditions	
Temperature range	25–45 °C (optimum 37 °C)
pH range	5.0–9.0 (optimum 7.0)
NaCl tolerance	0–7% (w/v)

**Table 3:** Comparison of protease production parameters before and after optimization

Parameter	Unoptimized	Optimized	Fold Increase	*p*-value
Protease activity (U/mL)	442 ± 15.2ᵇ	1152 ± 18.6ᵃ	2.61	< 0.001
Productivity (U/mL/h)	18.42 ± 0.63ᵇ	48.00 ± 0.78ᵃ	2.61	< 0.001
Biomass (g/L)	0.893 ± 0.025ᵇ	2.84 ± 0.09ᵃ	3.18	< 0.001
Yield coefficient (U/g)	494.96 ± 19.1ᵃ	405.63 ± 14.2ᵇ	0.82	< 0.001

Values represent means ± SD (n = 3). Different superscript letters within rows indicate statistically significant differences (p < 0.05, independent samples t-test)

#### Molecular identification

**Fig. 2:**
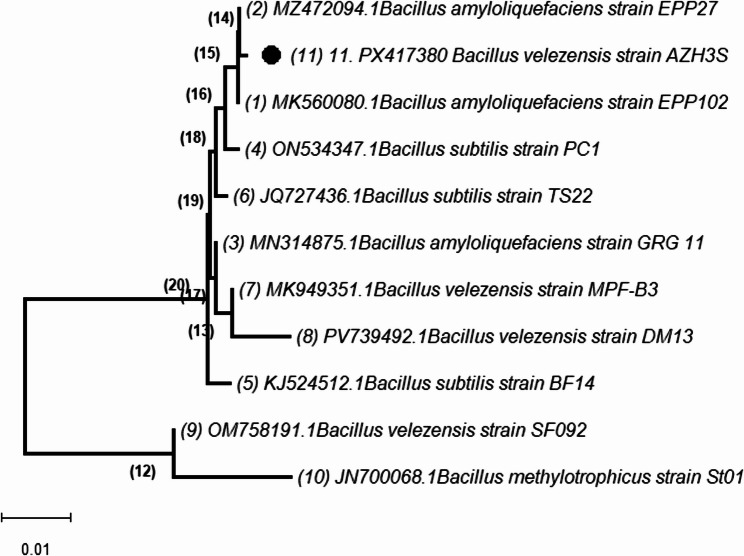
Phylogenetic Analysis of *Bacillus velezensis* AZH3S Based on 16 S rRNA Gene Sequence

Molecular analysis of the 16S rRNA gene sequence (1,456 bp) showed 99% sequence similarity to *Bacillus velezensis* strains in GenBank. Phylogenetic analysis using the neighbor-joining method confirmed clustering with *B. velezensis* clade which is rich in subtilisin-like and metalloprotease genes, confirming poultry waste as a high-value bioprospecting niche [41, 42] with highest similarity (99%) to *B. velezensis* strain MZ472094 (Fig. 2). The sequence was deposited in GenBank under accession number PX417380 and designated *Bacillus velezensis* AZH3S.

#### Optimization of culture conditions for protease production by *Bacillus velezensis* AZH3S

**Fig. 3:**
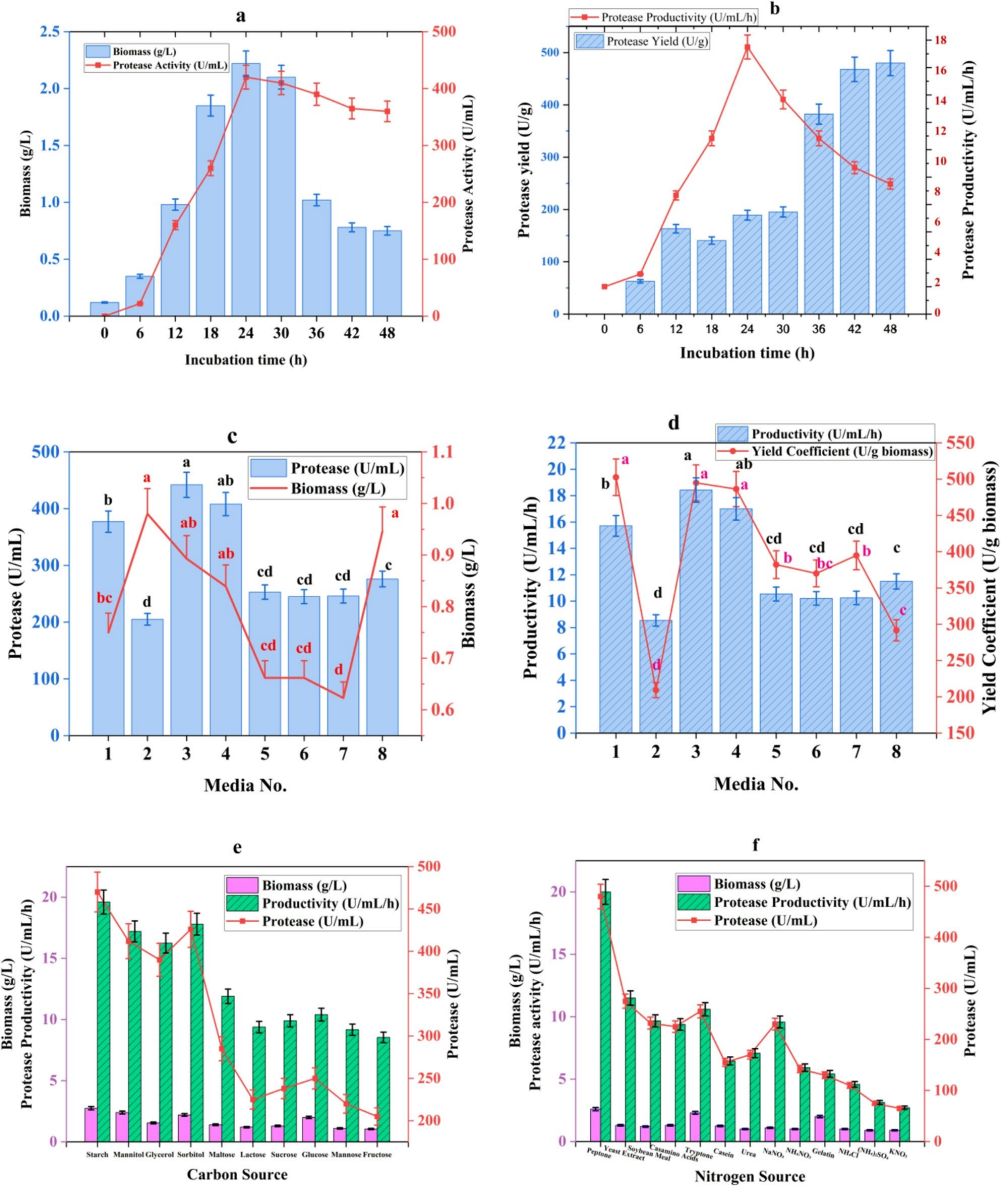
Optimization of culture conditions for protease production by *B. velezensis* AZH3S **A** Time-course profiles of biomass and protease production at 30 °C **B** Productivity and yield coefficient over time **C** Protease activity and biomass in different media (24 h, 37 °C) **D** Medium-dependent productivity and yield coefficient **E** Carbon source effects on protease biosynthesis and biomass accumulation **F** Nitrogen source effects on protease biosynthesis and biomass accumulation

Time-course analysis in casein–glucose medium at 37 °C revealed growth-associated protease synthesis (Fig. 3A). Biomass peaked at 2.22 g/L at 24 h before declining slightly. Protease activity was first detected at 6 h (22 U/mL) and reached maximum at 24 h (420 U/mL), corresponding to peak productivity (17.50 U/mL/h) and yield (189.19 U/g) (Fig. 3B). Protease levels remained > 85% of maximum through 48 h despite reduced biomass, indicating enzyme stability and continuous secretion. The yield coefficient increased during stationary phase to 480 U/g at 48 h, reflecting efficient synthesis under nutrient limitation [43, 44].

Protease production and biomass varied markedly across eight tested media (Fig. 3C, D). Medium 3 (glucose 10 g/L, casein 5 g/L, yeast extract 5 g/L) exhibited highest protease activity (442 U/mL), productivity (18.42 U/mL/h), and biomass (0.893 g/L). Medium 1 (gelatin 15 g/L, glycerol 3 mL/L, MgSO₄ 1.12 g/L) produced high activity (377 U/mL) and highest yield coefficient (502.67 U/g), attributed to glycerol bypassing CCR. Medium 2 (LB) yielded highest biomass (0.98 g/L) but lowest protease activity (205 U/mL), suggesting catabolite repression [43, 45].

Carbon source selection profoundly influenced protease biosynthesis (Fig. 3E). Starch was superior, supporting maximum protease activity (470 ± 1.1 U/mL), biomass (2.75 g/L), productivity (19.6 U/mL/h), and yield coefficient (170.90 U/g). Polyols performed well: sorbitol yielded 426 ± 1.9 U/mL with 193.60 U/g, while glycerol exhibited highest specific productivity (251.60 U/g). Monosaccharides proved least effective [44, 46].

Nitrogen source composition critically modulated protease biosynthesis (Fig. 3F). Peptone proved optimal, yielding maximum protease activity (480 ± 0.60 U/mL), biomass (2.60 g/L), productivity (20 U/mL/h), and yield coefficient (184.60 U/g). Yeast extract demonstrated exceptional yield coefficient (211.50 U/g). Inorganic nitrogen sources performed inadequately. Organic nitrogen sources’ superiority derives from their dual function as nutrient substrates and transcriptional inducers, triggering quorum-sensing cascades linked to protease secretion [2].

#### Statistical optimization of protease using BBD

Box-Behnken experimental design yielded protease activities ranging from 70 U/mL (Run 44: low starch, center peptone, neutral pH, center temperature, low agitation) to 1184.04 U/mL (Run 14: center starch, low peptone, neutral pH, low temperature, center agitation), demonstrating substantial impact of factor combinations (Table 4). Center point replicates (Runs 5, 15, 28, 30, 35, 43) exhibited excellent reproducibility (1160.24 ± 0.00 U/mL), confirming experimental precision and process stability. Model predictions showed strong concordance with experimental values, with residuals uniformly distributed between − 27.0 and + 25.96 U/mL (2.3% maximum deviation). Notable accuracy was observed at extreme conditions: Run 44 predicted 47.79 U/mL versus actual 70 U/mL, while Run 14 predicted 1174.39 U/mL versus actual 1184.04 U/mL. Close alignment between experimental and predicted values validates the model’s suitability for process optimization and industrial scale-up within investigated parameter ranges.

**Table 4:** Experimental design matrix with actual and predicted protease activity from Box-Behnken optimization by *Bacillus velezensis* AZH3S

Run	Starch (g/L)	Peptone (g/L)	pH	Temperature (°C)	Agitation (rpm)	Coded Levels (A, B, C, D, E)	Actual protease activity (U/mL)	Predicted protease activity (U/mL)
1	7.5	10	7	40	175	(0, + 1, 0, + 1, 0)	749.78	741.57
2	10	7.5	9	32.5	175	(+ 1, 0, + 1, 0, 0)	683.08	692.73
3	7.5	7.5	5	32.5	100	(0, 0, − 1, 0, − 1)	385.68	359.72
4	7.5	7.5	9	25	175	(0, 0, + 1, − 1, 0)	935.26	926.58
5	7.5	7.5	7	32.5	175	(0, 0, 0, 0, 0)	1160.24	1160.24
6	5	5	7	32.5	175	(–1, − 1, 0, 0, 0)	618.14	643.57
7	7.5	5	9	32.5	175	(0, − 1, + 1, 0, 0)	1136.48	1133.80
8	7.5	7.5	5	25	175	(0, 0, − 1, − 1, 0)	768.62	781.76
9	7.5	7.5	7	25	250	(0, 0, 0, − 1, + 1)	1046.60	1036.51
10	7.5	7.5	9	40	175	(0, 0, + 1, +1, 0)	674.06	668.50
11	10	7.5	7	40	175	(+ 1, 0, 0, + 1, 0)	889.16	874.57
12	5	7.5	5	32.5	175	(–1, 0, − 1, 0, 0)	438.42	420.36
13	10	7.5	7	32.5	100	(+ 1, 0, 0, 0, − 1)	470.88	470.58
14	7.5	5	7	25	175	(0, − 1, 0, − 1, 0)	1184.04	1174.39
15	7.5	7.5	7	32.5	175	(0, 0, 0, 0, 0)	1160.24	1160.24
16	10	7.5	5	32.5	175	(+ 1, 0, − 1, 0, 0)	953.88	949.49
17	7.5	10	7	32.5	250	(0, + 1, 0, 0, + 1)	590.44	599.70
18	5	10	7	32.5	175	(–1, + 1, 0, 0, 0)	503.58	521.48
19	7.5	7.5	7	25	100	(0, 0, 0, − 1, − 1)	221.18	248.59
20	7.5	5	7	32.5	100	(0, − 1, 0, 0, − 1)	445.70	428.54
21	10	7.5	7	25	175	(+ 1, 0, 0, − 1, 0)	792.10	790.21
22	7.5	10	9	32.5	175	(0, + 1, +1, 0, 0)	454.76	443.82
23	7.5	10	7	32.5	100	(0, + 1, 0, 0, − 1)	228.40	235.13
24	7.5	7.5	7	40	250	(0, 0, 0, + 1, +1)	573.62	563.57
25	5	7.5	7	32.5	250	(–1, 0, 0, 0, + 1)	690.30	688.93
26	7.5	5	7	32.5	250	(0, − 1, 0, 0, + 1)	997.56	982.93
27	7.5	10	7	25	175	(0, + 1, 0, − 1, 0)	556.80	539.05
28	7.5	7.5	7	32.5	175	(0, 0, 0, 0, 0)	1160.24	1160.24
29	7.5	7.5	5	32.5	250	(0, 0, − 1, 0, + 1)	738.92	727.09
30	7.5	7.5	7	32.5	175	(0, 0, 0, 0, 0)	1160.24	1160.24
31	5	7.5	7	25	175	(–1, 0, 0, − 1, 0)	770.42	777.93
32	10	7.5	7	32.5	250	(+ 1, 0, 0, 0, + 1)	727.86	748.40
33	7.5	7.5	7	40	100	(0, 0, 0, + 1, − 1)	405.10	432.54
34	7.5	5	7	40	175	(0, − 1, 0, + 1, 0)	683.00	682.88
35	7.5	7.5	7	32.5	175	(0, 0, 0, 0, 0)	1160.24	1160.24
36	10	5	7	32.5	175	(+ 1, − 1, 0, 0, 0)	1051.68	1050.94
37	5	7.5	9	32.5	175	(–1, 0, + 1, 0, 0)	743.60	739.59
38	7.5	7.5	5	40	175	(0, 0, − 1, + 1, 0)	734.58	750.85
39	7.5	10	5	32.5	175	(0, + 1, − 1, 0, 0)	802.96	814.24
40	7.5	5	5	32.5	175	(0, − 1, − 1, 0, 0)	681.36	700.91
41	7.5	7.5	9	32.5	100	(0, 0, + 1, 0, − 1)	294.80	298.85
42	5	7.5	7	40	175	(–1, 0, 0, + 1, 0)	409.78	404.59
43	7.5	7.5	7	32.5	175	(0, 0, 0, 0, 0)	1160.24	1160.24
44	5	7.5	7	32.5	100	(–1, 0, 0, 0, − 1)	70.00	47.79
45	7.5	7.5	9	32.5	250	(0, 0, + 1, 0, + 1)	832.26	850.43
46	10	10	7	32.5	175	(+ 1, + 1, 0, 0, 0)	604.66	596.38

Values represent means ± SD (n = 3). Center point conditions (runs 5, 15, 28, 30, 35, 43) were replicated six times to estimate pure error and assess model reproducibility

### Model development and validation

**Fig. 4:**
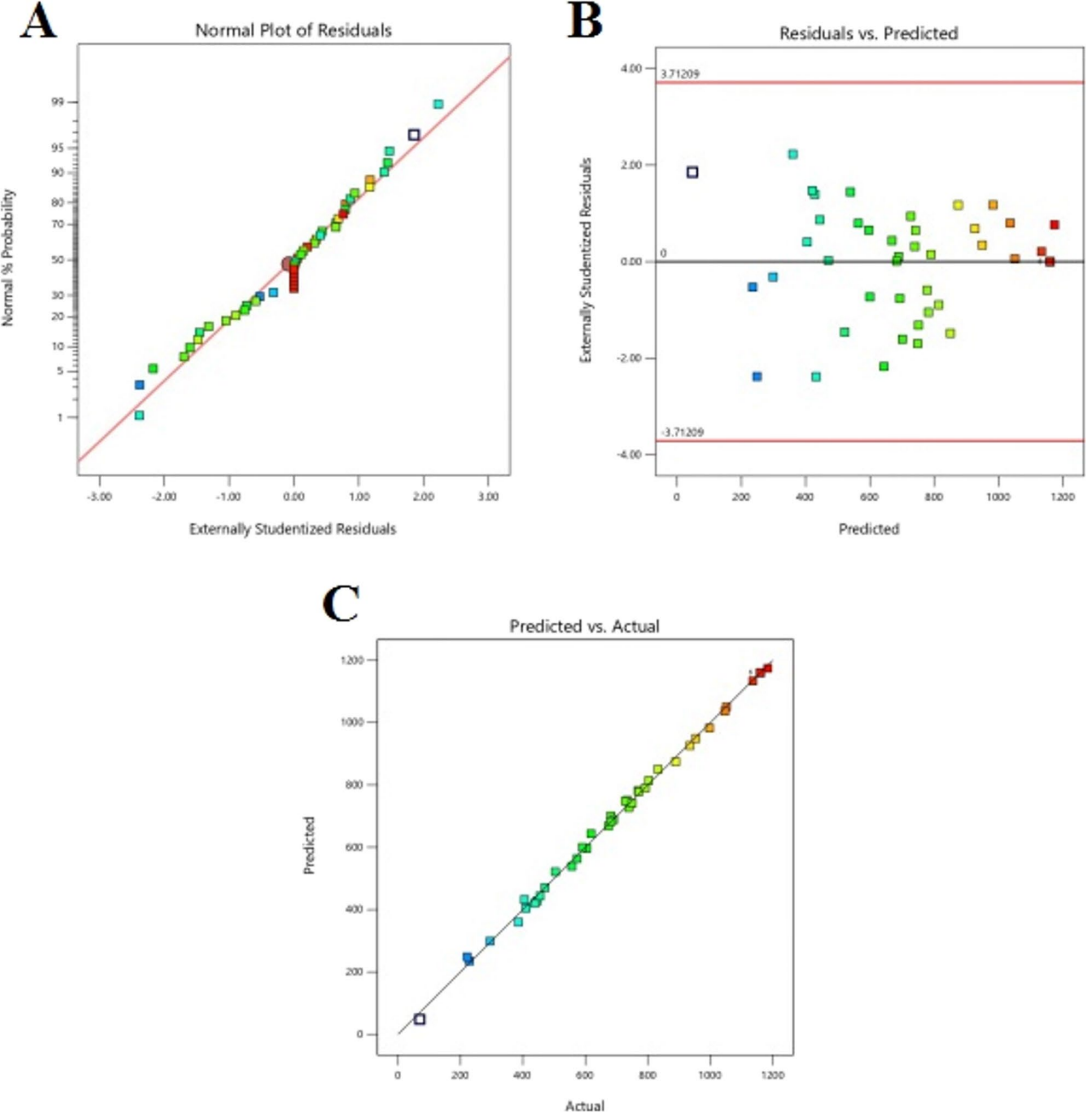
Model Diagnostic Validation for Box-Behnken Design. Normal probability plot (**A**), residuals versus predicted values (**B**), and predicted versus actual protease activity (**C**)

The quadratic regression model demonstrated exceptional statistical robustness (F = 607.69, *p* < 0.0001, R² = 0.9979), explaining 99.79% of response variability. Validation metrics confirmed model adequacy: adjusted R² (0.9963) and predicted R² (0.9918) showed close alignment (Δ < 0.2), indicating minimal overfitting, while adequate precision (93.975) substantially exceeded the threshold of 4, confirming excellent signal-to-noise ratio. Diagnostic analysis (Fig. 4) revealed normally distributed residuals, random error patterns without time-dependent effects, and tight predicted-versus-actual clustering, with externally studentized residuals ranging from − 2.38 to + 2.38.

### Effects of process variables and interactions

**Fig. 5:**
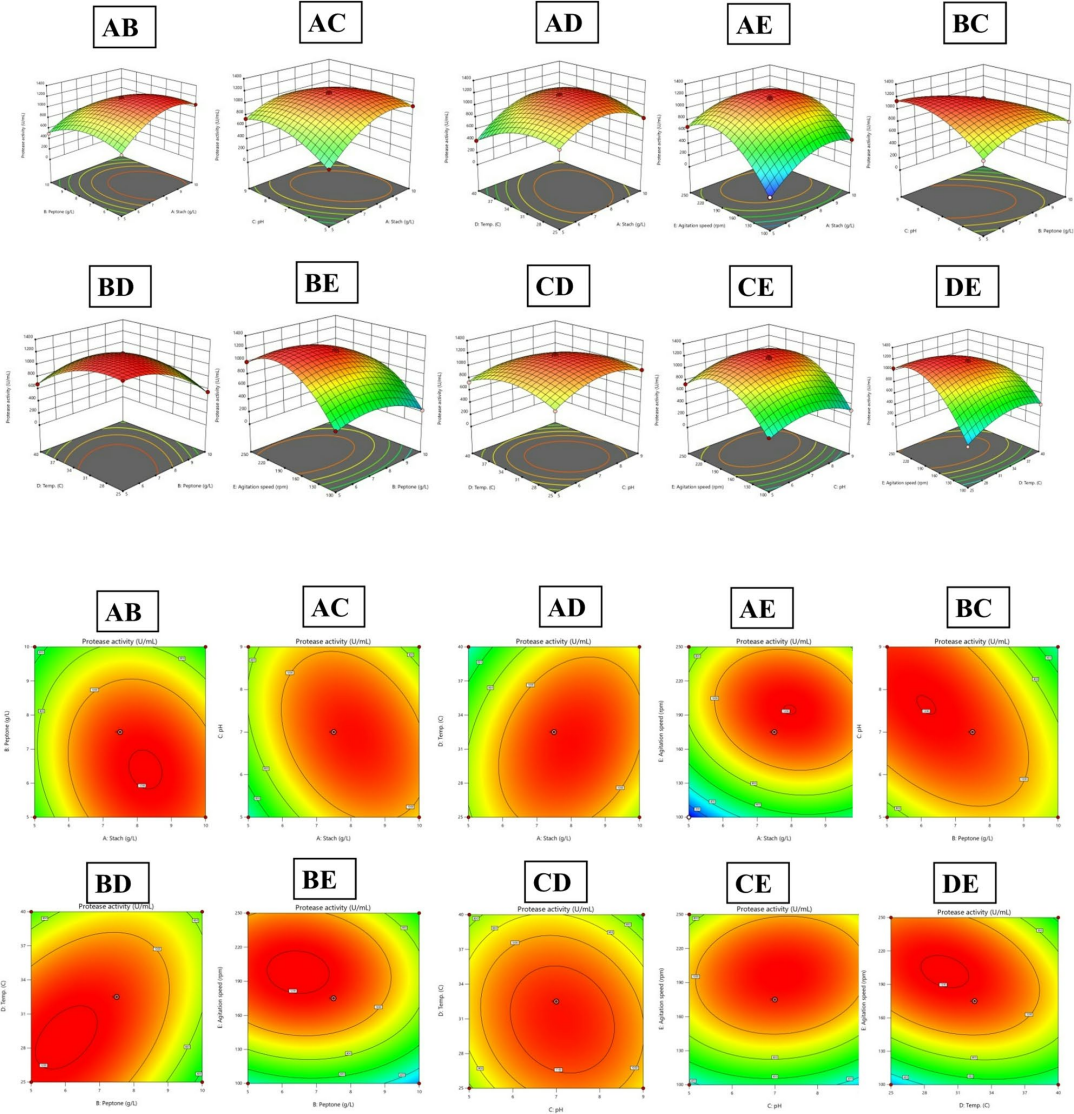
Response Surface and Contour Plots Illustrating Two-Factor Interactions. Three-dimensional surface plots (upper panels) and corresponding contour plots (lower panels) showing combined effects of (**A**) starch and peptone, (**B**) pH and temperature, and (**C**) agitation and starch on protease activity (U/mL)

All five variables significantly influenced protease activity (*p* < 0.05), with agitation speed exerting strongest positive linear effect (+ 229.74 U/mL), followed by starch concentration (+ 120.57 U/mL). Starch exhibited strong negative quadratic term (–264.90), indicating optimal activity within a narrow concentration range before viscosity-induced mass transfer limitations. Peptone showed negative linear (–144.16) and quadratic (–192.25) effects, consistent with nitrogen catabolite repression at elevated concentrations. Temperature demonstrated negative linear (–72.25) and quadratic (–183.52) responses, reflecting thermal enzyme stability trade-offs in mesophilic *Bacillus* strains. Fifteen two-factor interactions significantly affected protease biosynthesis, with peptone–pH (–200.83), peptone–temperature (+ 173.51), starch–pH (–144.00), and temperature–agitation (–164.22) showing most pronounced effects. Response surface analysis (Fig. 5) revealed maximum protease activity at intermediate starch with high agitation, while excessive peptone at alkaline pH suppressed production. Temperature–agitation interaction showed elevated agitation compensated for suboptimal temperatures by enhancing oxygen mass transfer [47–50].

#### Process optimization and experimental validation

**Fig. 6:**
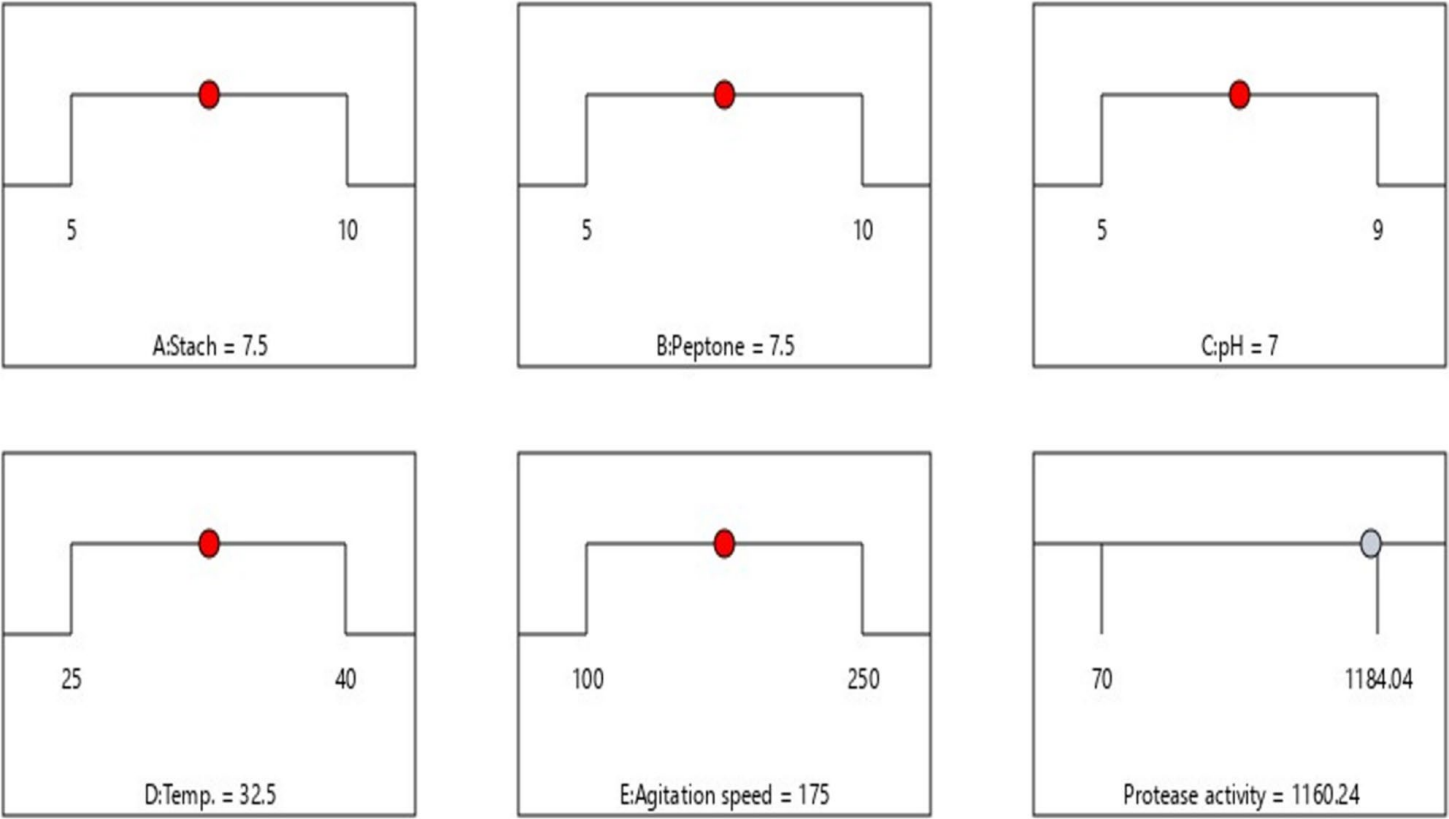
Optimization Ramp and Desirability Function Analysis. Individual parameter optimization profiles for starch (**A**), peptone (**B**), pH (**C**), temperature (**D**), and agitation speed (**E**) converging toward optimal conditions. Desirability = 1.000 achieved at predicted maximum protease activity of 1160.24 U/mL

Numerical optimization using desirability function analysis (Fig. 6) identified optimal conditions: starch 9.84 g/L, peptone 5.46 g/L, pH 7.5, temperature 33.1 °C, and agitation 244 rpm, predicting maximum protease activity of 1160.24 U/mL. Experimental validation yielded 1160.3 ± 9.2 U/mL (*n* = 3), representing 99.3% agreement and a 2.8-fold improvement over unoptimized production (442 U/mL). Statistical validation confirmed reliability: 95% CI 1145.32–1175.16 U/mL, 95% tolerance interval 1088.09–1232.39 U/mL, CV 2.44%, SD 17.74 U/mL, and no significant lack-of-fit (*p* > 0.05). The predictive equation in actual factors was:

Protease activity (U/mL) = − 14,945.3 + 871.75 A + 528.18B + 1,276.16 C + 164.855D + 41.2221E – 13.2984AB – 28.799AC + 6.10267AD – 0.484427AE – 40.166BC + 9.2536BD – 0.253093BE – 3.786CD + 0.307033CE – 0.291956DE – 42.3837 A² – 30.7597B² – 48.6999 C² – 3.26254D² – 0.0722521E².

The standardized quadratic form follows: **Y** = β₀ + β₁A + β₂B + β₃C + β₄D + β₅E + β₁₂AB + β₁₃AC + β₁₄AD + β₁₅AE + β₂₃BC + β₂₄BD + β₂₅BE + β₃₄CD + β₃₅CE + β₄₅DE + β₁₁A² + β₂₂B² + β₃₃C² + β₄₄D² + β₅₅E² + ε.

where A = starch, B = peptone, C = pH, D = temperature, E = agitation. The standardized quadratic form: Y = β₀ + Σβ_i_x_i_ + Σβ_i_ⱼx_i_xⱼ + Σβ_i__i_x_i_² + ε, where Y = protease activity, β₀ = intercept, β_i_ = linear coefficients, β_i_ⱼ = interaction coefficients, β_i__i_ = quadratic coefficients, ε = residual error. This model enables precise process control and provides a transferable framework for industrial protease optimization.

Values represent means ± SD (*n* = 3). Different superscript letters within rows indicate statistically significant differences (*p* < 0.05, independent samples t-test).

#### Protease purification and characterization

Extracellular protease was purified using a three-step protocol, achieving 5.82-fold purification with 32.0% yield (Table 5). From crude extract (895 mg total protein, 1.30 U/mg specific activity), ammonium sulfate precipitation (60–90% saturation) resulted in 1.71-fold purification with 56.0% recovery. Dialysis against 20 mM Tris–HCl buffer (pH 7.5) removed residual salts, increasing specific activity to 4.69 U/mg (3.61-fold purification) with 45.0% yield. Final Sephadex G-100 gel filtration yielded the most purified fraction (7.57 U/mg specific activity, 5.82-fold purification, 32.0% recovery). Activity losses during purification were attributed to: (i) incomplete protein resolubilization and co-precipitation during ammonium sulfate precipitation (44% loss), (ii) proteolytic autodegradation and membrane surface denaturation during dialysis (11% loss), and (iii) enzyme adsorption onto Sephadex G-100 matrix, dilution effects, and mechanical shear stress during gel filtration (13% loss). Despite cumulative losses reducing overall yield to 32%, the 5.82-fold increase in specific activity is essential for accurate biochemical characterization [19, 51, 52].

**Table 5:** Purification summary of extracellular protease from *Bacillus velezensis* AZH3S

Purification Step	Volume (mL)	Total Protein (mg)	Total Activity (U)	Specific Activity (U/mg)	Purification Fold	Yield (%)
Crude extract	500	895.00 ± 23.4ᵃ	1163 ± 42ᵃ	1.30 ± 0.05ᵈ	1.00ᵈ	100.0 ± 0.00ᵃ
(NH₄)₂SO₄ precipitation (60–90%)	115	292.10 ± 15.7ᵇ	651 ± 28ᵇ	2.23 ± 0.12ᶜ	1.71 ± 0.08ᶜ	56.0 ± 3.20ᵇ
Dialysis (20 mM Tris–HCl, pH 7.5)	39	111.40 ± 6.8ᶜ	523 ± 24ᶜ	4.69 ± 0.25ᵇ	3.61 ± 0.19ᵇ	45.0 ± 2.10ᶜ
Sephadex G-100 gel filtration	28	49.20 ± 3.1ᵈ	372 ± 19ᵈ	7.57 ± 0.38ᵃ	5.82 ± 0.29ᵃ	32.0 ± 1.80ᵈ

Values represent means ± SD (*n* = 3 independent purification batches).

#### Molecular weight determination

**Fig. 7:**
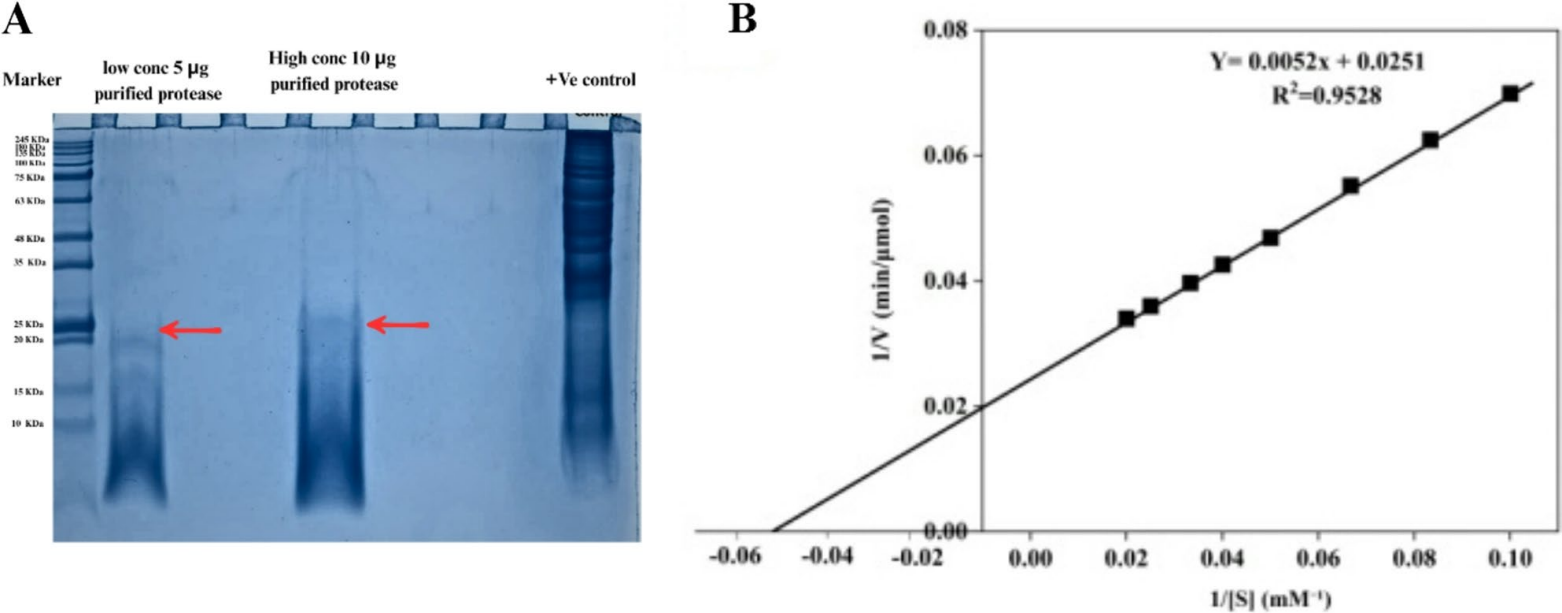
Molecular Weight and Kinetic Characterization of Purified Protease (**A**) SDS-PAGE analysis (**B**) Lineweaver-Burk double reciprocal plot

SDS-PAGE analysis revealed a single prominent band at approximately 20 kDa under denaturing conditions (Figs. 1S and 7A), confirming high purity following three-step purification. Lanes 2 and 3 (5 µg and 10 µg protein loading) showed concentration-dependent band intensity without contaminating proteins. The 20 kDa molecular weight is consistent with mature, processed serine proteases from the subtilisin family characteristic of *Bacillus* species. Low molecular mass combined with extracellular secretion indicates post-translational processing of the propeptide region, typical of subtilisin-like proteases. Absence of multiple bands confirms lack of proteolytic degradation during purification and storage, indicating enzyme stability under employed conditions [36, 50, 53–58].

### Kinetic properties of purified protease

Kinetic characterization using casein (0.1–2.5 mM) revealed classical Michaelis-Menten behavior without substrate inhibition or cooperative effects (Fig. 7B). Lineweaver-Burk analysis yielded a linear relationship (1/V = 0.0052[1/S] + 0.0251, R² = 0.9892), from which kinetic parameters were calculated: Km = 0.207 mM and Vmax = 39.8 µmol tyrosine equivalents/min. The low Km indicates exceptionally high substrate affinity, desirable for industrial applications requiring substrate economy. Compared to other *Bacillus* proteases, *B. velezensis* AZH3S exhibited the lowest Km (0.207 mM) and highest Vmax (39.8 µmol/min), outperforming *Bacillus* sp (Km = 4.60 mM, Vmax = 0.28.4 U/ml ) [59]‚ *B. licheniformis* NK (Km = 0.4384 mg/ml, Vmax = 88.57 U/ml) [60]‚ and *B. amyloliquefaciens* SP1 (Km = 0.125 mg/ml, Vmax of 12.820 mg/ml) [61]. These results demonstrate superior substrate affinity and catalytic efficiency, highlighting strong potential for industrial biocatalytic applications.

## Conclusion

This study isolated and identified *B. velezensis* AZH3S as a potent protease producer through targeted environmental screening and molecular characterization. Using Box–Behnken response surface methodology, five key fermentation parameters were optimized, resulting in a substantial increase in protease yield compared to the unoptimized condition. The predictive quadratic model highlighted agitation speed as the most influential factor, with significant two-factor interactions contributing to enhanced enzyme production. The purified protease also demonstrated superior kinetic performance relative to many reported *Bacillus* proteases. Overall, this work establishes a cost-effective and scalable process for sustainable industrial protease production. Future studies should explore heterologous expression systems and evaluate detergent compatibility to further expand the enzyme’s industrial applicability.

### Study limitations and future directions

Several limitations warrant consideration. First, optimization was conducted at laboratory scale (250 mL flasks), which may not represent oxygen transfer, mixing dynamics, and heat dissipation in industrial-scale bioreactors (> 1000 L). Second, comprehensive characterization of thermal stability (half-life at elevated temperatures), organic solvent tolerance, and resistance to surfactants and oxidizing agents remains necessary for detergent and industrial applications. Third, this study examined a single wild-type isolate; comparative analysis with additional *B. velezensis* strains may identify superior variants or synergistic co-culture strategies. Fourth, purification achieved only 5.82-fold enrichment with 32.0% yield, suggesting downstream processing optimization could enhance economic feasibility. Fifth, techno-economic analysis and life cycle assessment were not performed, limiting conclusions regarding commercial scalability and environmental sustainability. Finally, kinetic characterization determined Km and Vmax but not kcat or kcat/Km due to unknown enzyme molarity, limiting quantitative assessment of catalytic efficiency. Future studies should employ absolute protein quantification methods (amino acid analysis, analytical ultracentrifugation) to enable complete kinetic profiling including turnover number and catalytic efficiency.

## Supplementary Information

Supplementary Material 1

## Data Availability

The data used to support the findings of this study are available from the corresponding author upon request. *Bacillus velezensis* AZH3S was isolated from poultry waste sample and identified then deposited in NCBI GenBank with gene accession number PX417380.1 **https://www.ncbi.nlm.nih.gov/nuccore/PX417380**.
