# Supplementary material for: Harnessing Bacillus velezensis for enhanced protease biosynthesis: optimization, purification, and kinetic profiling
Source: BMC Microbiol. 2026 Mar 2;26:317. doi: 10.1186/s12866-026-04782-6 (PMC13063675; doi:10.1186/s12866-026-04782-6)
Supplement: Supplementary file 1 — Supplementary Material 1 [file 12866_2026_4782_MOESM1_ESM.docx]

**Harnessing *Bacillus velezensis* for Enhanced Protease Biosynthesis: Optimization, Purification, and Kinetic Profiling**

**Saied N. Fergany^1^, Salem S. Salem^1^*****[
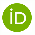
](https://orcid.org/0000-0003-2898-6708), Mohamed A. Abdel-Naby^2^, Tarek M Abdelghany^1^, Samah H. Abu-Hussien^3^***

**^1^**Botany and Microbiology Department, Faculty of Science, Al-Azhar University, Nasr City, Cairo, 11884, Egypt

**^2^**Chemistry of Natural and Microbial Products Department, National Research Centre, Dokki, Giza, Egypt

**^3^**Agricultural Microbiology Department, Faculty of Agriculture, Ain Shams University, Cairo, 11241, Egypt

*Corresponding authors: [salemsalahsalem@azhar.edu.eg](mailto:salemsalahsalem@azhar.edu.eg) (Scopus Author ID [57202162965](https://www.scopus.com/authid/detail.uri?authorId=57202162965)), [samah_hashem1@agr.asu.edu.eg](mailto:samah_hashem1@agr.asu.edu.eg)

**Table S1.** Box–Behnken Design Matrix for Optimization of Protease Production by *P15 isolate*

| **Run** | **Starch (g/L)** | **Peptone (g/L)** | **pH** | **Temperature (°C)** | **Agitation (rpm)** | **Coded Levels (A, B, C, D, E)** |
| --- | --- | --- | --- | --- | --- | --- |
| **1** | 7.5 (0) | 10 (+1) | 7 (0) | 40 (+1) | 175 (0) | (0, +1, 0, +1, 0) |
| **2** | 10 (+1) | 7.5 (0) | 9 (+1) | 32.5 (0) | 175 (0) | (+1, 0, +1, 0, 0) |
| **3** | 7.5 (0) | 7.5 (0) | 5 (–1) | 32.5 (0) | 100 (–1) | (0, 0, –1, 0, –1) |
| **4** | 7.5 (0) | 7.5 (0) | 9 (+1) | 25 (–1) | 175 (0) | (0, 0, +1, –1, 0) |
| **5** | 7.5 (0) | 7.5 (0) | 7 (0) | 32.5 (0) | 175 (0) | (0, 0, 0, 0, 0) |
| **6** | 5 (–1) | 5 (–1) | 7 (0) | 32.5 (0) | 175 (0) | (–1, –1, 0, 0, 0) |
| **7** | 7.5 (0) | 5 (–1) | 9 (+1) | 32.5 (0) | 175 (0) | (0, –1, +1, 0, 0) |
| **8** | 7.5 (0) | 7.5 (0) | 5 (–1) | 25 (–1) | 175 (0) | (0, 0, –1, –1, 0) |
| **9** | 7.5 (0) | 7.5 (0) | 7 (0) | 25 (–1) | 250 (+1) | (0, 0, 0, –1, +1) |
| **10** | 7.5 (0) | 7.5 (0) | 9 (+1) | 40 (+1) | 175 (0) | (0, 0, +1, +1, 0) |
| **11** | 10 (+1) | 7.5 (0) | 7 (0) | 40 (+1) | 175 (0) | (+1, 0, 0, +1, 0) |
| **12** | 5 (–1) | 7.5 (0) | 5 (–1) | 32.5 (0) | 175 (0) | (–1, 0, –1, 0, 0) |
| **13** | 10 (+1) | 7.5 (0) | 7 (0) | 32.5 (0) | 100 (–1) | (+1, 0, 0, 0, –1) |
| **14** | 7.5 (0) | 5 (–1) | 7 (0) | 25 (–1) | 175 (0) | (0, –1, 0, –1, 0) |
| **15** | 7.5 (0) | 7.5 (0) | 7 (0) | 32.5 (0) | 175 (0) | (0, 0, 0, 0, 0) |
| **16** | 10 (+1) | 7.5 (0) | 5 (–1) | 32.5 (0) | 175 (0) | (+1, 0, –1, 0, 0) |
| **17** | 7.5 (0) | 10 (+1) | 7 (0) | 32.5 (0) | 250 (+1) | (0, +1, 0, 0, +1) |
| **18** | 5 (–1) | 10 (+1) | 7 (0) | 32.5 (0) | 175 (0) | (–1, +1, 0, 0, 0) |
| **19** | 7.5 (0) | 7.5 (0) | 7 (0) | 25 (–1) | 100 (–1) | (0, 0, 0, –1, –1) |
| **20** | 7.5 (0) | 5 (–1) | 7 (0) | 32.5 (0) | 100 (–1) | (0, –1, 0, 0, –1) |
| **21** | 10 (+1) | 7.5 (0) | 7 (0) | 25 (–1) | 175 (0) | (+1, 0, 0, –1, 0) |
| **22** | 7.5 (0) | 10 (+1) | 9 (+1) | 32.5 (0) | 175 (0) | (0, +1, +1, 0, 0) |
| **23** | 7.5 (0) | 10 (+1) | 7 (0) | 32.5 (0) | 100 (–1) | (0, +1, 0, 0, –1) |
| **24** | 7.5 (0) | 7.5 (0) | 7 (0) | 40 (+1) | 250 (+1) | (0, 0, 0, +1, +1) |
| **25** | 5 (–1) | 7.5 (0) | 7 (0) | 32.5 (0) | 250 (+1) | (–1, 0, 0, 0, +1) |
| **26** | 7.5 (0) | 5 (–1) | 7 (0) | 32.5 (0) | 250 (+1) | (0, –1, 0, 0, +1) |
| **27** | 7.5 (0) | 10 (+1) | 7 (0) | 25 (–1) | 175 (0) | (0, +1, 0, –1, 0) |
| **28** | 7.5 (0) | 7.5 (0) | 7 (0) | 32.5 (0) | 175 (0) | (0, 0, 0, 0, 0) |
| **29** | 7.5 (0) | 7.5 (0) | 5 (–1) | 32.5 (0) | 250 (+1) | (0, 0, –1, 0, +1) |
| **30** | 7.5 (0) | 7.5 (0) | 7 (0) | 32.5 (0) | 175 (0) | (0, 0, 0, 0, 0) |
| **31** | 5 (–1) | 7.5 (0) | 7 (0) | 25 (–1) | 175 (0) | (–1, 0, 0, –1, 0) |
| **32** | 10 (+1) | 7.5 (0) | 7 (0) | 32.5 (0) | 250 (+1) | (+1, 0, 0, 0, +1) |
| **33** | 7.5 (0) | 7.5 (0) | 7 (0) | 40 (+1) | 100 (–1) | (0, 0, 0, +1, –1) |
| **34** | 7.5 (0) | 5 (–1) | 7 (0) | 40 (+1) | 175 (0) | (0, –1, 0, +1, 0) |
| **35** | 7.5 (0) | 7.5 (0) | 7 (0) | 32.5 (0) | 175 (0) | (0, 0, 0, 0, 0) |
| **36** | 10 (+1) | 5 (–1) | 7 (0) | 32.5 (0) | 175 (0) | (+1, –1, 0, 0, 0) |
| **37** | 5 (–1) | 7.5 (0) | 9 (+1) | 32.5 (0) | 175 (0) | (–1, 0, +1, 0, 0) |
| **38** | 7.5 (0) | 7.5 (0) | 5 (–1) | 40 (+1) | 175 (0) | (0, 0, –1, +1, 0) |
| **39** | 7.5 (0) | 10 (+1) | 5 (–1) | 32.5 (0) | 175 (0) | (0, +1, –1, 0, 0) |
| **40** | 7.5 (0) | 5 (–1) | 5 (–1) | 32.5 (0) | 175 (0) | (0, –1, –1, 0, 0) |
| **41** | 7.5 (0) | 7.5 (0) | 9 (+1) | 32.5 (0) | 100 (–1) | (0, 0, +1, 0, –1) |
| **42** | 5 (–1) | 7.5 (0) | 7 (0) | 40 (+1) | 175 (0) | (–1, 0, 0, +1, 0) |
| **43** | 7.5 (0) | 7.5 (0) | 7 (0) | 32.5 (0) | 175 (0) | (0, 0, 0, 0, 0) |
| **44** | 5 (–1) | 7.5 (0) | 7 (0) | 32.5 (0) | 100 (–1) | (–1, 0, 0, 0, –1) |
| **45** | 7.5 (0) | 7.5 (0) | 9 (+1) | 32.5 (0) | 250 (+1) | (0, 0, +1, 0, +1) |
| **46** | 10 (+1) | 10 (+1) | 7 (0) | 32.5 (0) | 175 (0) | (+1, +1, 0, 0, 0) |


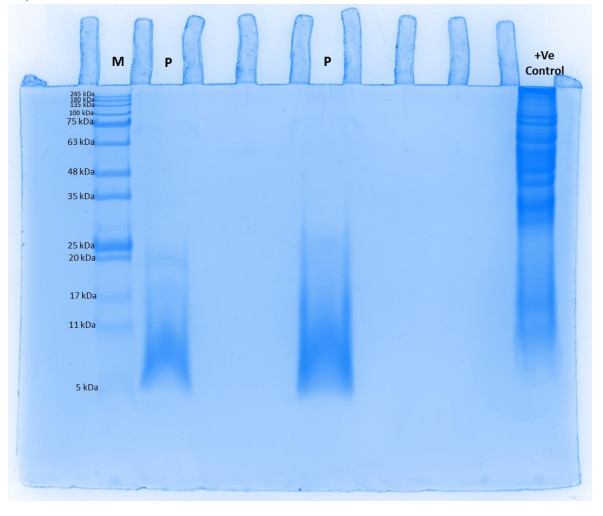


**Figure 10A S**. SDS-PAGE stained with Coomassie blue.

M: Marker

P: Samples P (Protease) (first: low conc 5μg purified protease, Second: high conc 10μg purified protease)
